# Progress on diagnostic and prognostic markers of pancreatic cancer

**DOI:** 10.32604/or.2023.028905

**Published:** 2023-04-10

**Authors:** HONG YANG, WAN LI, LIWEN REN, YIHUI YANG, YIZHI ZHANG, BINBIN GE, SHA LI, XIANGJIN ZHENG, JINYI LIU, SEN ZHANG, GUANHUA DU, BO TANG, HONGQUAN WANG, JINHUA WANG

**Affiliations:** 1 The State Key Laboratory of Bioactive Substance and Function of Natural Medicines, Beijing, 100050, China; 2 Key Laboratory of Drug Target Research and Drug Screen, Institute of Materia Medica, Chinese Academy of Medical Science and Peking Union Medical College, Beijing, 100050, China; 3 Department of Pancreatic Cancer, Tianjin Medical University Cancer Institute and Hospital, National Clinical Research Center for Cancer, Tianjin’s Clinical Research Center for Cancer, Key Laboratory of Cancer Prevention and Therapy, Tianjin, 300060, China

**Keywords:** Pancreatic cancer, Biomarkers, Liquid biopsy, Systematic review

## Abstract

Pancreatic cancer is a malignant disease characterized by low survival and high recurrence rate, whose patients are mostly at the stage of locally advanced or metastatic disease when first diagnosed. Early diagnosis is particularly important because prognostic/predictive markers help guide optimal individualized treatment regimens. So far, CA19-9 is the only biomarker for pancreatic cancer approved by the FDA, but its effectiveness is limited by low sensitivity and specificity. With recent advances in genomics, proteomics, metabolomics, and other analytical and sequencing technologies, the rapid acquisition and screening of biomarkers is now possible. Liquid biopsy also occupies a significant place due to its unique advantages. In this review, we systematically describe and evaluate the available biomarkers that have the greatest potential as vital tools in diagnosing and treating pancreatic cancer.

## Introduction

The pancreas is mainly composed of three main functional compartments, the ducts, the exocrine glands and the endocrine glands. The ducts and exocrine glands are the main sites where pancreatic cancer (PC) occurs. Pancreatic ductal adenocarcinoma (PDAC) accounts for more than 90% of pancreatic cancers, which are characterized by high heterogeneity, insidious onset, aggressiveness, rapid development, difficult detection, and poor prognosis [1]. Other pancreatic tumors in the exocrine compartment include pancreatic acinar cell carcinoma (PACC), pancreatoblastoma, and solid pseudo-papillary neoplasm (SPN), among others. They are rare and share common features of cellar tumors with little intervening stromal and abnormal beta-catenin expression. Pancreatic tumors in endocrine glands are generally defined as pancreatic neuroendocrine tumors (PanNET) or islet cell tumors, which are less malignant and much rarer, constituting less than 3% of all primary pancreatic cancers [2].

The global incidence of PC as one type of digestive tract cancer has been increasing year by year, and still exhibits the highest mortality rate and the lowest survival rate among all cancers. Over 80% of pancreatic cancer patients are at the advanced stage when they are first diagnosed, and surgery is suitable for only 10% of patients. In addition, these patients often go on to have postoperative metastasis and chemoradiotherapy resistance, resulting in high recurrence and mortality rates. At present, the diagnosis and treatment of PC faces many challenges. There are no obvious symptoms at the early stages. PC has no specific clinical manifestations or tumor markers, and its imaging characteristics are not typical. The pathogenesis and disease progression are complicated because PC is a highly heterogeneous and complex disease with persistent genomic instability that can promote progression of the cancer. Gene rearrangements varied greatly from patient to patient in terms of types and numbers, half of which occurred in the early stages of tumor progression [3], which makes it even harder to diagnose and treat. The tumor’s anatomical position is deep in the posterior side of the right upper abdomen of the human body, which makes it more difficult to detect. There are also difficulties in distinguishing PC from chronic pancreatitis by conventional ultrasound and other examination methods. Therefore, it is particularly important to identify and discover key biomarkers for early diagnosis of PC.

A biological marker (biomarker) is an objectively measured and evaluated characteristic molecule that is an indicator of normal biological or pathological processes, or of pharmacological responses to therapeutic interventions, which is critical for the diagnosis and prognosis of cancer patients. Currently used biomarkers include those for the diagnosis and prognosis of tumors such as alpha-fetoprotein (AFP) for liver cancer and cytokeratin (CYFRA) 21-1 for non-small cell lung cancer. Biomarkers are also of great importance for the early detection and prognosis of pancreatic cancer, and this paper reviews the progress and current status of biomarkers for diagnosis and prognosis of PC.

## Biomarkers for Diagnosis and Prognosis of Pancreatic Cancer

## Proteomic Biomarkers

### Carbohydrate antigen (CA)

CA19-9 is a modified Lewis(a) blood group antigen, which is produced by exocrine epithelial cells. It is generally bound onto the surface of erythrocytes and acts as a component of glycoproteins and mucins. It is the only FDA-approved clinical PC biomarker that has been applied broadly in the diagnosis of PDAC. Elevated levels of CA19-9 indicate progression, increased likelihood of recurrence, and poor prognosis of PC patients [4]. Pleskow et al. [5] first reported that CA19-9 could be a prospective biomarker in PDAC. In that study, among the 261 patients enrolled (54 PDAC), the results showed that CA19-9 had 70% sensitivity, 87% specificity, 59% positive predictive value, 92% negative predictive value and 84% accuracy (with a cut-off value of 70 U/mL), regardless of cancer stage. In addition, preoperative CA19-9 levels have been shown to indicate whether a PDAC is resectable or not. For example, a CA19-9 level > 300 U/mL, would indicate that a patient was in an advanced stage of disease. However, it was reported that only one out of three patients had non-resectable cancers (CA19-9 > 300 U/mL). Thus, the study recommended that staging laparoscopy should be performed on patients with CA19-9 > 130 U/mL. Postoperative measurements of CA19-9 can also be used to provide prognostic information on patients with localized disease, as a low postoperative CA19-9 level was related to improved overall survival [6].

However, there are some limitations to the validity of CA19-9 [7]. The positive rate in patients with resectable PCa is only 65%. The utility of screening asymptomatic populations is questionable, and PC and chronic pancreatitis cannot be effectively distinguished. Also, elevation of CA19-9 occurs in many other malignant tumors or diseases including biliary obstruction. Therefore, CA19-9 is usually combined with computed tomography (CT) and magnetic resonance imaging (MRI) for the diagnosis, but not for the screening due to inadequate sensitivity and specificity.

Other carbohydrate antigens include CA50, CA242 and CA125. Although none of them are FDA-approved as biomarkers in PDAC, they have very good potential as diagnostic markers. Most of the research on CA50 was reported in the 1980s, and those studies showed a diagnostic performance similar to that of CA19-9 with 46–78% sensitivity and 70–95% specificity; but CA50 was prone to false positives in benign cholestasis jaundice and liver parenchymal disease [8]. Wu et al. [9] reported that the best results with CA 50 as a diagnostic marker in 48 cancer patients and 93 healthy controls were 71% sensitivity and 93% specificity. CA 242 is a sialylated glycosphingolipid antigen that acts as a glycoprotein/glycolipid on the cell surface or as an O-linked glycoprotein (mucin) in serum. CA 242 is always co-expressed with CA50, but the two are recognized by different monoclonal antibodies. Although CA242 has similar specificity to CA19-9 and CA50 in the diagnosis of pancreatic cancer, its sensitivity was lower compared to theirs [10]; therefore, strategies for combined marker detection could be adopted. CA125 is a tumor marker, first identified as a product of ovarian carcinoma cells. Haglund [11] measured the serum concentration of CA125 in 95 PC patients and 106 patients with benign pancreatic, biliary and hepatocellular diseases, and compared it with the levels of CA19-9 and CEA (carcinoembryonic antigen). Results showed that there were elevated CA125 levels (>35 U/mL) in 45% of the PC patients; but, elevated CA125 levels were also detected in 24% of benign diseases, suggesting a potential problem with false-positive results.

Considering the limited effectiveness and the false positive or negative results of diagnosis with markers alone, the combination of CA19-9 and other markers to increase the accuracy has become the preferred goal in future research [12–14]. The details are shown in Table 1.

**Table 1:** Selected combinations of CA19-9 with other biomarkers

Combination of biomarkers	Sensitivity	Specificity	Pub. date	Cohorts
CA19-9+CA125+LAMC2	84.7	89.2	2014	PDAC *vs.* benign controls
CA 19-9+CA 242	89	75	2015	PC *vs.* benign and healthy controls
CA 19-9+CA 125+CEA+CA242	90	94	2015	PC *vs.* healthy controls
CA19-9+5MC+H2A1.1+H2AZ+H3K4Me2	92	90	2015	PC *vs.* benign and healthy controls
CA 19-9+albumin+IGF-1	94	95	2016	PDAC *vs.* CHP and healthy controls
CA 19-9+MUC5A	75	83	2017	PC *vs.* CHP and benign controls
CA 19-9+CEA+HGF+OPN+ctDNA	64	99.5	2017	PDAC *vs.* healthy controls
CA 19-9+THBS2	87	98	2017	PDAC *vs.* benign and healthy controls
CA 19-9+MDMs	92	92	2021	PDAC *vs.* healthy controls

Note: PDAC, pancreatic ductal adenocarcinoma; PC, pancreatic cancer; CHP, chronic pancreatitis; benign, benign pancreatic disease.

### Carcinoembryonic antigen-related cell adhesion molecules (CEACAMs)

CEACAMs belong to the glycosylphosphatidylinositol (GPI)-linked immunoglobulin (Ig) superfamily. In the CEACAM family, different isoforms are similar in structure but differ in their expression pattern. Among them, CEACAM5 and CEACAM1 have been more frequently studied in PDAC [15].

CEACAM5 (CEA) is a 180–200 kDa glycoprotein, which is derived from the endoderm epithelial tissue and is primarily found in the gastrointestinal, urinary, and respiratory tracts. It is one of the best known members of the Ig superfamily and is widely used clinically in the diagnosis of digestive system tumors. In 30–60% of PC patients, serum CEA level was increased, which suggests its diagnostic value. Although CEA as a diagnostic marker has a lower sensitivity and specificity than CA19-9, CEA is undoubtedly an important marker for the group with little or no secretion of CA19-9 (5–10%). Compared with CA19-9, a high level of CEA can also act as a more reliable independent predictor of advanced PDAC with an odds ratio of 4.21 (*p* = 0.001) [16]. The preoperative serum panel of CEA^+^/CA125^+^/CA19-9 (value ≥ 1000 U/mL) helped to pick out a subgroup of patients with poor outcomes after surgery [17]. Thus, the diagnostic effectiveness of CEA in PC remains unclear. Hence, a comprehensive analysis is necessary to evaluate the application of CEA in the diagnosis and prognosis of PC patients in clinics.

CEACAM1 is highly expressed in PDAC. The diagnostic sensitivity and specificity of CEACAM1 in differentiating PDAC from chronic pancreatitis were better than those of CA19-9. Moreover, the diagnostic accuracy of combining CEACAM1 with CA19-9 was significantly improved [18]. The expression of CEACAM1 was also assessed in pancreatic intraepithelial neoplasia (PanIN), and the researchers found a statistically significant difference in CEACAM 1 expression between normal pancreatic ducts and those from patients with different degrees of PanIN (*p* < 0.001) [19]. Thus, CEACAM1 may serve as a promising indicator for PC and precancerous lesions in the pancreas, but needs further validation studies.

### Mucins (MUCs)

Mucins (MUCs) are a family of high molecular weight, multifunctional glycoproteins that are mainly distributed on the surface of gastrointestinal epithelial cells and exert crucial roles in gut lubrication and protection. MUC1, MUC3, MUC4, MUC5AC, MUC5B, MUC6, MUC7, MUC13, MUC16 and MUC17 are expressed in PC, and MUC5B and MUC13 are also expressed in normal pancreatic tissue. Among them, MUC1, MUC5AC, MUC4 and MUC16 are abnormally expressed in the early stages of PanIN, and the expression increases as the cancer progresses through carcinogenesis and tumor invasion [20]. Therefore, MUCs may serve as specific biomarkers to discriminate among PC, PanIN and pancreatitis and be used for early diagnosis of PC. For instance, MUC1 is highly expressed in over 60% of PC patients and is negatively correlated with tumor size and patient prognosis. MUC4 and MUC16 could be used to diagnose PDAC patients with high specificity, and their sensitivities were 63% and 67%, respectively. The cystic fluid MUC4 also has high sensitivity in accurately distinguishing between high- and low-risk cystic tumors in patients with intraductal papillary mucinous tumors (IPMN) [21].

The high expression of MUCs is also considered to be an independent adverse factor for the prognosis of pancreatic cystic tumors. Some pathologists believe that pancreatic tumors expressing mucin are a distinct category. By evaluating the clinical and epidemiological features of PDAC cases, Crippa et al. [22] found that the 5-year disease-specific survival rate of patients without MUCs expression was almost 100%, and that the 5-year OS of invasive mucinous cystic tumors, main-duct IPMNs, branch-duct IPMNs and combined IPMNs patients expressing MUCs were 58%, 51%, 56% and 64%, respectively. Therefore, these characteristics of MUCs may be useful in preoperative differentiated diagnosis and clinical management strategies. In addition, MUCs are also included with other biomarkers such as the combination of CA 19-9 and MUC5A to improve PC diagnosis rate, sensitivity and specificity [13].

### Receptor tyrosine kinases (RTKs)

Membrane proteins are often used as drug targets in targeted therapy. At the same time, the fact that they are frequently secreted into the blood makes them potentially useful as biomarkers for early detection. Many classical receptor tyrosine kinases (RTKs) are overexpressed in PC, suggesting good potential as biomarkers.

Epidermal growth factor receptor (EGFR) is overexpressed in up to 90% of PDAC and its upregulation may be related to more aggressive tumor behavior and a higher recurrence rate. Erlotinib (EGFR tyrosine kinase inhibitor) has been approved to treat PC, and the subpopulation of patients who may be more likely to respond to erlotinib treatment can be identified by predictive biomarkers of PDAC, such as the EGFR ligand, angiogenin (ANG) [23].

Insulin-like growth factor (IGF) binding protein (IGFBP), a member of the IGF family, has been proven to be capable of distinguishing patients with early stages of PDAC from healthy individuals. A three-point diagnostic panel of CA19-9, IGFBP2, and IGFBP3 was more effective than CA19-9 alone [24], which implicates IGFBP2 and IGFBP3 as promising compensatory biomarkers for CA19-9. Another study showed that the combination of IGFBP and mesothelin (MSLN), a cell surface glycoprotein, was effective in diagnosing PDAC. Although neither IGFBP2 nor MSLN reached the diagnostic accuracy of CA 19-9, they alone or in combination could correctly identify 18 of the 28 samples which were not identified by CA 19-9. In the age-adjusted model, IGFBP2 (*p* = 0.36) and MSLN (*p* = 0.29) were not significant subsets for predicting survival. To sum up, serum IGFBP2 and MSLN were inaccurate classifiers individually, but their combination may be useful in diagnosis [25]. Moreover, in PDAC, high expression of IGF-1 and IGF1R was also related to high tumor grade and low survival in PDAC patients, which indicates some prognostic value [26].

Vascular endothelial growth factor-A (VEGF-A) was overexpressed in both PDACs and serous cystic tumors (SCN), which is related to high microvessel density and disease progression. It was reported that VEGF-A as a biomarker for the diagnosis of SCN had 100% sensitivity, 97% specificity, and a critical value of 8500 pg/mL, which helped to differentiate between SCN and precancerous/malignant pancreatic cysts, and afforded an opportunity for early detection, prevention, and cure [27].

### Macrophage inhibitory cytokine-1 (MIC-1)

Macrophage inhibitory cytokine-1 (MIC-1) is a member of the transforming growth factor-β superfamily, which was originally found in activated macrophages. MIC-1 is highly expressed in several cancer types including PDAC [28]. In a meta-analysis of fourteen studies comprising 2826 subjects in total, the results showed that the diagnostic accuracy of MIC-1 was comparable to CA19-9 for PC with a sensitivity of 80% *vs.* 71% and a specificity of 85% *vs.* 88% [29]. Given that MIC-1 had only 63.1% sensitivity in detecting patients with CA19-9-negative PDAC, MIC-1 combined with CA19-9 may make the diagnostic efficiency better. The serum MIC-1 level was remarkably decreased in PDAC patients following curative resection, but returned to high levels when the malignancy relapsed, indicating that MIC-1 has a role in determining the prognosis or monitoring the progress of PDAC therapy [30].

### Glypican-1 (GPC1)

Glypican-1 (GPC1), a membrane-anchored protein, has been reported to be abnormally expressed in various cancers and possibly involved in tumorigenesis; therefore, it is looked upon as a potential clinical biomarker in the blood. Overexpression of GPC1 has been observed in both PC cell lines and tissues, and its expression level was closely related to the pathological grade and clinical stage of the cancer, and higher levels were associated with poor patient prognosis, suggesting its diagnostic and prognostic impact [31]. Melo et al. [32] used flow cytometry to detect GPC1^+^ exosomes in the serum of PDAC patients, chronic pancreatitis patients, and healthy individuals. They found that all 190 patients’ serum GPC1^+^ exosomes were higher than those of healthy controls, with an almost perfect diagnostic value (~100% sensitivity and ~100% specificity). Moreover, GPC1^+^ exosomes in serum were independent disease-specific prognostic markers, and their prognostic value was higher than that of CA19-9. Qian et al. [33] reported similar results, that advanced stage PC patients had higher GPC1+ EVs than healthy controls (*p* < 0.01). The patients whose GPC1+ EVs levels decreased significantly after regional intra‑arterial chemotherapy (RIAC) treatment experienced increased OS rates. In spite of these results, using GPC1 as a biomarker is still controversial. Zhou et al. [34] stated that, although the serum GPC1 level was reported to be a prognostic indicator, it was not an ideal diagnostic biomarker for PDAC patients. GPC1 is not a tissue-specific protein, and the GPC1 expressed by cancerous tissue may be confused with normal secretions from healthy tissues. Also, although serum does not contain fibrinogen and other components, it does contain many active clotting factors, and each factor is a highly active protease. It is not clear whether any of them might cut GPC1, leading to false results. Therefore, the application of serum GPC1 in diagnosis needs to be further validated.

### Osteopontin (OPN)

Osteopontin is a phosphorylated glycoprotein produced by osteoblasts, arterial smooth muscle cells, various epithelial cells, activated T cells and macrophages and secreted into most body fluids. It can influence the invasiveness of PC cells and its overexpression is related to lower survival rates in cancer patients [8]. Koopman et al. [35] reported 80% sensitivity and 97% specificity of serum OPN in discriminating between patients with resectable pancreatic cancer and healthy controls, which is better than CA19-9, where the sensitivity was 62%. Rychlíková et al. [36] demonstrated that higher OPN levels (>102 ng/ml) could be a prospective diagnostic biomarker to differentiate between PC and chronic pancreatitis. However, the diagnostic accuracy of OPN combined with CA19-9 was not very high; but, a diagnostic panel composed of OPN, tissue inhibitor of metalloproteinases-1 (TIMP-1) and CA19-9 showed better sensitivity and specificity [37]. Further studies are needed to validate its clinical value.

### Secreted protein acidic and rich in cysteine (SPARC)

SPARC, also called osteonectin or basement membrane protein 40 (BM40), is an extracellular matrix glycoprotein, which is involved in multiple biological processes, such as wound repair, tissue remodeling, morphogenesis and cell differentiation. In pancreatic cancer, SPARC has been identified as a common site for aberrant methylation and a mediator of tumor-stromal interaction. Han et al. [38] performed a meta-analysis on 1623 patients in 10 studies and found that high SPARC expression, especially in the stroma, indicated poor outcomes for PDAC patients. Interestingly, SPARC expressed in peritumoral fibroblasts has been shown to be correlated with a worse long-term prognosis in PC patients [39], which may partly explain the results of Han et al. Additionally, SPARC can also be used as a predictive biomarker in Nab-paclitaxel therapy. Compared with the low SPARC group, the high SPARC group had excellently longer median overall survival after treatment with albumin paclitaxel (17.8 months *vs.* 8.1 months) [40].

### S100 calcium-binding protein family

S100 proteins are members of a superfamily of small EF-hand Ca^2+^-binding proteins that are mostly found in PDAC, PanIN and IPMN rather than in normal pancreatic ductal cells [41]. The S100 protein family contains over 20 members, each of which is encoded by a different gene. Among them, S100A2, S100A6 and S100P are important PC markers. S100A2 is useful for predicting the outcome of pancreatectomy. S100A2-negative PDAC patients had positive survival benefits from pancreatectomy even with non-clear surgical margins or lymph node metastasis. S100A6 is composed of 90 amino acids and its expression is absent during the PanIN period. However, with the progress of PanIN, the nuclear expression level of S100A6 protein gradually increases, which is an indicator of poor patient prognosis. This suggested that, although S100A6 was expressed in early pancreatic cancer, nuclear S100A6 could serve as an independent prognostic factor [42]. S100P is a 95-amino-acid protein, whose expression is positively correlated with the progression of PanIN, indicating that S100P is essential to the progression from PanIN to invasive ductal adenocarcinoma. Ohuchida et al. [43] found that the S100P levels were noticeably higher in patients with IPMN and PC than in non-neoplastic pancreas tissues. A sensitivity of 85% and specificity of 77% were obtained to diagnose stage 0/IA/IB/IIA of PDAC with an AUC of 0.82, indicating that S100P could be effective for early diagnosis.

### Tumor M2 pyruvate kinase (Tu M2-PK)

Tu M2-PK is a pyruvate kinase isoenzyme, which is one of the key rate-limiting enzymes in glycolysis. Ventrucci et al. [44] found that Tu M2-PK in serum was a powerful diagnostic tool due to its higher serum levels in advanced PDAC and chronic pancreatitis (ChP) compared to healthy controls. Unfortunately, Tu M2-PK was not fit for early PDAC detection because there were no differences in Tu M2-PK levels between early PDAC and ChP. However, some studies have reported that the Tu M2-PK level in plasma was related to the stage of pancreatic cancer. Subsequently, Goonetilleke et al. reported the parameters of Tu M2-PK in plasma as a diagnostic test for PC: the area under the curve (AUC) was 0.623, the critical cut-off value was 27 U/mL, sensitivity was 66%, and specificity was 58%. Elevated Tu M2-PK (>27U/mL) was significantly correlated with an adverse prognosis and metastatic disease. In all PDAC tests, the sensitivity and specificity of Tu M2-PK were lower than those of CA19-9, which may be related to the fact that the Tu M2-PK level is not subject to Lewis’s phenotype or cholestasis. But, Tu M2-PK combined with CA19-9 can significantly enhance diagnostic sensitivity [45]. Bandara found another benefit of Tu M2-PK as a marker in that an elevated preoperative level portended a worse prognosis and survival rate in patients with periampullary malignancy and PDAC [46].

## Genetic Markers

Due to the continuing progress in nucleic acid sequencing technology, the genetic changes in PDAC have been well characterized. KRAS, TP53, SMAD4 and CDKN2A were dominant mutations in PDAC and the rate of each mutation in PC patients was more than 50%. The mutation frequencies of the four genes varied at different stages of PanIN, which suggested that identifying gene mutations may be a useful tool for accurately distinguishing early invasive carcinoma from low or high-grade dysplasia. In addition, some genes with mutation rates of 5–10% have been discovered including *RNF43, KDM6A, BCORL1, RBM10, MLL3 (KMT2C), ARID1A, TGFBR2, MAP2K4, ATM*, and *SMARCA4*. Although the incidence of most mutations is less than 5%, they are closely related to molecular subtypes of PDAC [47].

### KRAS

*KRAS* mutations occur in 90–95% of PCs and are common in precancerous and precursor lesions, such as PanINs and IPMNs. Among *KRAS* mutations, the most common are transformations from wild-type GGT to GAT (G12D, aspartic acid), GTT (G12V, valine), CGT (G12R, arginine), TGT (G12C, cysteine) and GCT (G12A, alanine). These mutations are at codon 12 of exon 2, while other point mutations at codons 11, 13, 61, or 146 are less frequent [48]. Different mutations are enriched in different subtypes. For example, the G12R mutation was enriched in classical subtypes, while G12D and G12V were enriched in basal-like subtypes, suggesting that *KRAS* mutation has different roles in the progression and indirect differentiation of tumors [49]. The expression of mutant *KRAS* gene in stromal, non-glandular and basal-like subtypes was high, and was closely related to an extremely aggressive tumor type and undifferentiated phenotypes of PDAC histology. Also, an increase in *KRAS* mutation was enough to induce basal-like characteristics [50], which suggested that different *KRAS* mutations could be used to distinguish molecular phenotypes of PDAC.

*KRAS* mutations are often related to low overall survival (OS) regardless of the stage of PDAC, suggesting the potential of *KRAS* mutations as prognostic markers. It is worth noting that the subtypes of *KRAS* mutation also have different effects on the prognosis of PDAC patients. For example, the OS of PDAC patients with the G12D mutation (6 months) was significantly decreased compared to patients with the G12R mutation (14 months), G12V mutation (9 months) and wild type (9 months) (HR = 1.47; *p* = 0.003), irrespective of chemotherapy [51]. In conclusion, since *KRAS* mutation is so common in PDAC, the detection of *KRAS* mutation in fluid or tumor tissue can have an important impact on the diagnosis, prognosis evaluation and treatment decision of PDAC.

### TP53

*TP53* inactivation mutations exist in 50–75% of pancreatic cancer, which inhibits the recognition of DNA damage and prevents the cell cycle arrest that can enable cells to avoid cell cycle checkpoints and apoptosis signals. In 2013, the prevalence of *TP53* mutations in precursors and PDAC was demonstrated by Kanda et al. using resected specimens [52]. The mutation rates of *TP53* in low-grade PanIN/IPMN, high-grade PanIN/IPMN and PDAC were 5.4%, 42.8% and 75%, respectively, which were in rough agreement with the prevalence detected in tissue samples. By using a novel next-generation sequencing method, PDAC and IPMN cases could be distinguished even at low levels (0.1–1%) of *TP53* and/or *SMAD4* mutations in duodenal fluid, with 32.4% sensitivity and 100% specificity [53]. In addition, 89% sensitivity and 100% specificity for the diagnosis of high-grade IPMN and invasive IPMN were obtained by combining *KRAS*/*GNAS* mutation and *TP53/PIK3CA/PTEN* alterations [54].

### CDKN2A

One of the most commonly mutated genes in PC is *CDKN2A*, which is also known as *CDK4I*, *p16-INK4a* or *MTS-1* and is mutated and inactivated in 98% of sporadic PC. *CDKN2A* can hinder the formation of the cyclinD-CDK4/CDK6 complexes at the G1/S checkpoint thereby regulating the cell cycle, and its inherited modification is related to a high risk of pancreatic cancer [55]. Evidence showed that *CDKN2A* mutations cold induce more aggressive PC, suggesting its potential as a prognostic marker. Studies have indicated that the OS of patients with a *CDKN2A* mutation was shorter, and that the mutation rate was negatively correlated with the survival rate [56]. A clinical study also revealed a predictive role of *CDKN2A* in the surgical treatment PC. In that study, 88 patients with PC were divided into a surgical resection group (69 patients) and a non-resection group (19 patients). The mutation rates of *CDKN2A*, 3′-UTR C580T, in the two groups were different, that is, the proportions of CC genotype (81% and 19%), CT genotype (0% and 63%), and TT genotype (26% and 11%), in the two groups were different. The results showed that the disease-free survival rate for the CC genotype was significantly different from that of the CT or TT genotypes (*p* = 0.039) [57].

### SMAD4

*SMAD4* is inactivated in about 55% of pancreatic cancers and acts as a tumor suppressor gene. Homozygous deletion or mutation can result in the frequent deletion of *SMAD4*, which in turn reduces the SMAD4-dependent inhibitory effect on TGF-β and promotes atypical TGF-β signaling, thereby contributing to pro-tumorigenic responses and poor prognosis [58–59]. The RTOG 1201 radiotherapy oncology trial further validated the value of *SMAD4* as a prognostic marker by evaluating the response of radiotherapy to *SMAD4* status in PC patients at the locally advanced stage [60]. It is worth mentioning that *SMAD4* mutation often occurs at the late stage of pancreatic cancer [47], therefore, *SMAD4* mutation may be used for the diagnosis of advanced precancerous lesions. Furthermore, *SMAD4* levels were obviously higher in classical tumors than in basal-like subtype PDX tumors, which may suggest *SMAD4* as a potential biomarker for distinguishing different molecular subtypes of PDAC [49].

### BRCA1/BRCA2

*BRCA1* (RNF53) is a tumor suppressor, which encodes a protein with E3 ubiquitin-protein ligase activity. The protein can mediate the formation of lys-6-linked polyubiquitin chains, thereby participating in response to DNA damage. *BRCA2* (also known as *FANCD1*) activates the RAD51 recombinase, which can engage in double-strand break repair, cytokinesis and cell death [54]. The mutation rates of *BRCA1* and *BRCA2* in familial pancreatic cancers are <1.2% and 5–17%, respectively, and those mutations increase the risk of disease by 2.26-fold and 3.5 to 10-fold. Most of these mutations lead to the dysfunction of the proteins they encode, which promotes tumorigenesis [61]. It has been suggested that universal genetic testing of all PC patients to enable rapid disease detection and develop individualized treatment plans is particularly important. Most studies also assessed *BRCA1*/*BRCA2* as prognostic and predictive biomarkers, but there have been mixed results. Among 71 *BRCA*-positive PDAC patients, a much better prognosis was obtained in *BRCA*-mutated patients compared to the normal PDAC population, while a study by Blair et al. [62] showed the opposite results. It compared PDAC patients whose *BRCA1/2* was mutated with age-matched controls and found lower OS and disease-free survival (DFS) in patients with *BRCA1/2* mutations [63]. Therefore, the impact of *BRCA* mutation on the prognosis of pancreatic cancer requires further evaluation. From a predictive perspective, advanced PDAC patients with *BRCA1/BRCA2* mutations showed superior OS after treatment with cisplatin than patients without *BRCA1/BRCA2* mutations [64]. Another study reported that the 617delT *BRCA2* mutation could prolong survival after chemotherapy in a patient with advanced pancreatic cancer, despite the unfavorable prognosis [65]. Taken together, these studies suggest that *BRCA1/BRCA2* mutations may serve as good predictive biomarkers for the response of pancreatic tumors to chemotherapeutic agents related to DNA damage.

### RNF43

Among PDACs, 5–10% have been proven to have an *RNF43* mutation. *RNF43* encodes a transmembrane protein with ubiquitin E3 ligase activity and its N-terminal domain can induce ubiquitination of frizzled receptor 5 (FZD5), thereby inhibiting the FZD5 receptor-dependent Wnt signaling cascade and exerting a tumor suppressor effect. In PDAC, low RNF43 levels and an increased expression of frizzled receptors occurred, which promoted Wnt signaling activity and cell proliferation, leading to tumor transformation [66]. Yu et al. [53] performed NGS of pancreatic cyst fluid and identified *RNF43* mutations as biomarkers in pancreatic cystic neoplasms (PCNs). By contrast, Sakamoto et al. [67] reported that *RNF43* alteration was not associated with the malignancy of IPMN. Therefore, the role of *RNF43* in the diagnosis of PDAC needs further study.

### GNAS

*GNAS* is considered an oncogene, mutated in ~4% of PC patients. Mutation at codon 201 of *GNAS* was only observed in IPMNs with a mutation rate of 41–75%, leading to sustained activation of a heterotrimeric Gs protein (GSP) and an increased level of cyclic adenosine monophosphate glycoside (cAMP), which activates protein kinase A (PKA) with subsequent cancer-promoting activity [68]. Correspondingly, GSP encoded by *GNAS* was present in IPMN but not in associated adenocarcinoma. These results suggest the importance of *GNAS* mutation in early precancerous pancreatic lesions. By analyzing *GNAS* mutation in circulating cell‑free DNA (cfDNA) from 57 patients with pancreatic cystic neoplasms (PCNs) (including 34 IPMN patients), Hata et al. [69] found that the positive rate of *GNAS* mutation in cfDNA was significantly higher in IPMN patients than in other PCN patients (32% *vs.* 0%, *p* = 0.002). These results suggested that mutation of *GNAS* in cfDNA could be a novel tool to classify cyst types and identify the intestinal subtype of IPMN from other PCNs. *GNAS* mutation was associated with tumorigenesis rather than progression and survival outcomes [70]. This implies that *GNAS* mutation is not suitable as a prognostic marker.

### GATA6

*GATA6* has a proposed oncogenic function due to being selectively amplified in some pancreatic cancers. This amplification was observed as an outcome of continual genomic copy number gain. The Wnt antagonist Dickkopf-1 (DKK1) can be negatively regulated by the transcription factor GATA6, which can then promote the activation of the canonical Wnt signaling pathway and accelerate the progression of PanIN to PC [71]. In spite of these findings, the role of GATA6 in PC is still controversial. Martinelli et al. [72] reported that GATA6 could inhibit the epithelial-mesenchymal transition (EMT) *in vitro* and cell dissemination *in vivo* by regulating transcription factors such as FOXA1/2 directly or indirectly. This inhibitory effect of GATA6 was also validated in PDAC genetic mouse models [73]. Therefore, what role GATA6 plays in PC and its impact on prognosis is still unclear, and whether it can be used as a prognostic marker needs to be further explored. Nonetheless, the value of *GATA6* mutation as a diagnostic and predictive marker has been revealed. Aung et al. [74] identified *GATA6* as a biomarker of the classical subtype of PC in a prospective clinical trial driven by genomic molecular profiling (COMPASS). Zhong et al. [71] reported that *GATA6* could be a predictive marker of response to adjuvant chemotherapy of PC.

### HER2

*HER2* (*NEU*/*ERBB2*) belongs to the ErbB family that acts as an oncogene by encoding a membrane-bound tyrosine kinase. This protein can activate multiple signaling cascades by forming homologous or heterodimeric complexes, which promotes tumor cell growth, metastasis, and chemotherapy resistance. *HER2* gene amplification and protein expression have been reported to occur in 2.1–24% and 7.2–61.2% of PCs, respectively [75]. *HER2* as a diagnostic and prognostic marker has been reported in breast cancer and gastric cancer, but whether it can be a prognostic marker for PC is still unknown. *HER2* amplification was confirmed to be unrelated to the outcomes of patients by Sharif et al. [76] and Stoecklein et al. [77] In contrast, Komoto et al. [78] demonstrated that overexpression of *HER2* could result in significantly shorter survival times.

### BRAF^V600E^ mutation

*BRAF* is an oncogene and *BRAF*^*V600E*^ mutation occurs frequently. The mutation is closely associated with mismatch repair deficiency (MMR-D) and CpG island methylation, as well as high microsatellite instability (MSI-H) phenotype through hypermethylation of the promoter region of the *MLH1* gene [79]. Although *KRAS* mutations commonly occur in PCs, wild-type *KRAS* tumors also exist. Those *KRAS*-WT PDAC patients often have other oncogenic mutations, for instance, a *BRAF* mutation. The *BRAF*^*V600E*^ mutation occurred in 3% of PCs and was incompatible with *KRAS* mutations [80]. Studies have reported that *BRAF*^*V600E*^ was significantly bound up with survival outcomes in MMR-D colorectal cancer patients and may be used as a predictor of the duration of the immune checkpoint inhibitor response. There have been few studies on the role of *BRAF* as a marker in pancreatic cancer, but it is foreseeable that BRAF is of great significance for guiding the diagnosis, prediction/prognosis and treatment of *KRAS*-WT patients.

## Epigenetic Markers

Epigenetics is the heritable alteration of gene expression without changes in the DNA sequences of the genes, which can ultimately lead to transformations of phenotype. It includes DNA methylation, genomic imprinting, histone modification, and editing of non-coding RNAs (ncRNAs). The pathogenesis of PC and the proliferation, metastasis and drug resistance of PC cells are not only regulated by genetic mutations but also profoundly affected by epigenetic changes [81], thus, epigenetic modifications could be used for diagnosis and prediction/prognosis of PC. Among the known epigenetic alterations, aberrant DNA methylation has been studied the most. Methylation patterns can be relatively easily detected by methylation-specific PCR (MS-PCR) analysis.

Abnormal DNA methylation occurs frequently in PC, affecting many promoters and CpG islands. Sato et al. [82] identified 475 candidate genes with DNA methylation in four PC cell lines. Among them, the *UCHL1*, *CLDN5*, *NPTX2* and *SFRP1* genes were found to be hypermethylated in most primary PCs. On the contrary, hypomethylation of the promoters of claudin4, *LCN2*, 14-3-3r (*SFN*), *TFF2*, *S100A4* and mesothelin were reported in PDAC [83]. Given that many genes exhibit proportionally high aberrant methylation in PC that can be detected by MS-PCR, abnormally methylated genes may serve as sensitive markers for the diagnosis of PC. For example, glycine N-methyltransferase (GNMT), which is involved in regulating methylation and folate metabolism, is frequently hypermethylated in PDAC samples compared with healthy pancreatic tissue. GNMT methylation as a diagnostic biomarker has achieved great diagnostic parameters with 90% sensitivity and 80% specificity [84]. Yokoyama et al. [85] found abnormal expression and promoter hypomethylation of MUC4 in PanINs and PDACs, but not in healthy pancreatic tissue by testing pancreatic tissue samples from 57 PC patients and 98 controls. Furthermore, this hypomethylation in PDAC patients was profoundly correlated with poor prognosis. Thus, a selected aberrant methylation group could likely be used for diagnostic purposes. A clinical trial showed that a test group consisting of five CpG sites had the potential to diagnose PDAC patients with 51% sensitivity, 90% specificity, and an AUC of 0.76. These sites were in the interleukin 10 (*IL10*, P348F), lipocalin 2 (*LCN2*, P86R), T-cell acute leukemia 1 (*TAL1*, P817F), zeta chain associated kinase (*ZAP70*, P220), and absent in melanoma 2 (*AIM2*, P624) genes [86]. Moreover, some abnormally methylated genes may play an essential part in the occurrence and development of PC, and thus can be used as prognostic markers. For instance, Reprimo (*RPRM*) is a gene involved in G2 cell cycle arrest induced by TP53. It was found that *RPRM* was methylated in 60% of PCs and its methylation was strongly related to genetic instability and unfavorable prognosis in PDAC patients following surgical resection. DNA methylation as a biomarker is superior to genetic and serum markers in many ways [80]. Abnormal DNA methylation of specific CpG islands in cancer cells occurs at a higher rate than genetic deficiencies and can be detected with good sensitivity, even when embedded in a large amount of normal DNA because the MS-PCR technique for detecting abnormal DNA methylation is relatively simple and accurate. Abnormal methylation often occurs in tumors at an early stage resulting in the loss or activation of key signaling pathways, thus, abnormal DNA methylation can be served as a good diagnostic or prognostic biomarker of cancer.

Histones and their modifications are critical to the structure of chromatin. There is a pair of reversible enzymes, histone deacetylase (HDAC) and histone acetyltransferase (HAT), participating in regulation of the majority of histone modifications. When their regulatory action is disturbed, it can induce alterations in chromatin structure that lead to the occurrence of diseases including malignant tumors. Trimethylated histone H3 at lysine 27 (H3K27me3), which is catalyzed by polycomb repressive complex 2 (PRC2), is one of the most common histone methylation modifications. By analyzing 165 PDAC samples, Wei et al. [87] found that patients with low H3K27me3 had a lower five-year survival rate than patients with high H3K27me3 (11% *vs.* 23%). Furthermore, multivariate analysis revealed that H3K27methylation could act as an independent prognostic factor for the OS of PDAC patients when combined with tumor size and lymph node status. Histones could also be used as predictors of PDAC treatment outcomes. However, in PDAC patients receiving adjuvant chemotherapy, there was no association between low H3K4me2, H3K4me3, or H3K9me2 levels and patient survival [88].

Although numerous markers of epigenetic alterations for the diagnosis and prognosis of PC have been discovered, most markers have only been tested in a small group of patient samples, so further validation is needed before these markers can be formally used in the clinic.

## Liquid Biopsy

Liquid biopsy is an emerging tool for detection and diagnosis of diseases, which can be used for early screening, identifying subtypes, guiding treatment plans, and monitoring the effect of treatment and recurrence in cancer patients by collecting blood samples, detecting biomarkers and performing tumor analysis. Liquid biopsy has many unique advantages [89]. Any fluid from the body, such as urine, blood, or spinal fluid can potentially be sampled and used for screening. Peripheral blood has been most extensively used for analyses based on circulating tumor cells (CTCs) or circulating tumor-derived, cell-free DNA (ctDNA), which can provide biological information to predict cancerous or precancerous conditions. Liquid biopsy circumvents the difficulty of getting surgical specimens or biopsy tissue for detection. Without the need for surgical or invasive procedures, multiple samples can be obtained over time, reflecting real-time dynamic changes in the tumor. Therefore, liquid biopsy is being applied more and more to the diagnosis, prediction and prognosis of PDAC. At present, liquid biopsy is mainly performed to detect CTCs, ctDNA, ncRNA and exosomes which are derived from serum or plasma.

### Circulating tumor cells (CTCs)

CTC is the general term for tumor cells in the peripheral blood, which facilitate the metastatic migration of solid tumors to distant sites. Some studies have found that both the subtypes and the total counts of CTCs in the peripheral blood of patients with PDAC were upregulated compared with healthy individuals [90]. The detection of tumor markers on the surface of CTCs or the substrates inside CTCs, such as epithelial markers, mRNA and DNA mutations, can enable real-time biopsy of cancer. Mataki et al. measured CEA mRNA of internal substrates of CTCs by qRT-PCR, with sensitivity and specificity of 33.3–75% and 94.6–96% for PC diagnosis, respectively, which had higher diagnostic values than CEA and CA 19-9. Ankeny et al. separately analyzed *KRAS* mutations in CTCs and primary tumor tissues from five PDAC patients and found 100% consistency between both results [91]. CTCs also have clinical value in prognosis through prediction of PC metastasis. Poruk et al. [92] concluded that epithelial markers, such as cytokeratin, and cancer stem-cell markers, ALDH, CD133, CD44, on the surface of CTCs in the blood were independently predictive of poor survival and cancer recurrence.

However, PDAC has been found to produce only low numbers of CTCs, which would make it an extremely challenging process to achieve efficient isolation and enrichment of CTCs in future samples. In addition, the detection performance of CTCs is largely dependent on the methods, so it is urgent to establish a standardized testing procedure and conduct a large-scale validation to prove its diagnostic accuracy before using it in the clinic.

### Circulating tumor DNA (ctDNA)

Normally, DNA exists only in the nucleus, but it can be released from cells as a result of apoptosis or necrosis and circulate in the blood as free DNA (cfDNA) that can be identified by assay. In cancer patients, the DNA released by tumor cells, which is highly fragmented, is called ctDNA (circulating tumor DNA) and has an average size of 170 base pairs [93]. The selected ctDNAs reflect tumor heterogeneity and have a strong correlation with tumor burden. The ctDNA assay is currently being investigated as a tumor-specific biomarker test that yields information on tumor state and genetics as well as prognostic value.

In PDAC, ctDNA can be derived from tumor cells or CTCs and has shown great potential in screening patients with PDAC. CTCs are already isolated from most such patients for the purpose of identifying KRAS mutations. Compared with sequencing genomic DNA from tumor tissue, the time needed for ctDNA was significantly shorter (11 *vs.* 33 days; *p* < 0.0001) and the trial enrollment rate was greatly improved (9.5 *vs.* 4.1%; *p* < 0.0001) for ctDNA detection and genotyping [94]. Although ctDNA was generally less sensitive than CA19-9 for the early diagnosis of PC, its combination with CA 19-9 tremendously increased the diagnostic sensitivity to 91% [95]. It was also confirmed that ctDNA combined with CA19-9, CEA, HGF, OPN or other protein biomarkers could also be used for early diagnosis. In terms of prognostic value, ctDNA has also been proven to be related to a lower OS and a higher risk of tumor recurrence in PC patients [96]. The combination of ctDNA with mutant KRAS is a good indicator for monitoring the progression of PC during or after chemoradiotherapy treatment or surgery, which may be more suitable for predicting PDAC progression than CA19-9 and CTCs [97].

Overall, ctDNA detection can provide real-time, comprehensive disclosure of systemic multi-lesion mutation information using a noninvasive method. Additionally, given that the methylation of DNA is an early event in oncogenesis, the DNA methylation pattern can also be used as a diagnostic marker in the early stages of disease. However, PDAC had the lowest ctDNA detection rate (83.4%) compared to other cancers [96], which signifies the need for further research on how to improve the enrichment and detection rate of ctDNA.

### Non-coding RNAs (ncRNAs)

Non-coding RNAs are a class of RNA without protein-coding function or an open reading frame (ORF), but which can be reverse transcribed from protein-coding genes. Many studies have found that ncRNA profiles are tumor-specific, particularly miRNA and lncRNA profiles. They can be readily detected in the blood of patients and used as potential diagnostic biomarkers for malignancies including PDAC [90].

### MicroRNAs (miRNAs, miRs)

MicroRNAs are small, endogenous, non-coding, single-stranded RNAs that regulate gene expression by mediating translation inhibition and/or degradation of homologous mRNAs. They have no ORF and can be integrated into nanoparticles or bound to the human Argonaute-2 (hAgo2) protein to prevent degradation by RNases. Some miRNAs, such as miR-10, miR-21, miR-155 and miR-196, have been shown to be abnormally expressed in PC and other disease states [58]; circulating miRNAs in the plasma or serum of PC patients, like miR-486-5p, miR-1290, or miR-100a, showed superior diagnostic value over CA19-9 [90]. Moreover, miRs in stool and urine specimens were also used for diagnosis. In urine samples, miR-223 and miR-204 can discriminate early-stage cancer from chronic pancreatitis. The levels of miR-21 and miR-155 in stool specimens of PDAC patients were higher than that of controls [98]. Similarly, Lai et al. [99] measured the changes in levels of various miRNAs from a cohort of 29 PDAC patients before and after surgical resection. They identified an exosomal miRNA signature, including miR10b and miR30c, which could effectively distinguish PDAC from chronic pancreatitis. The combined panel of miRs could also be used for diagnostic purposes. Schultz et al. [100] tested another panel of miRNAs (miR-145, miR-150, miR-223, and miR-636) and found that it had great diagnostic value. The diagnostic sensitivity and specificity of both miR panels were 85%, but the sensitivity and specificity were not superior to CA19-9.

Panels of miRs also have the potential to act as prognostic biomarkers. High concentrations of miR-21, miR-155 and miR-203 and low concentrations of miR-34a were associated with lower OS and disease-free survival (DFS) in PDAC patients [101]. Low levels of miR-494 and miR-218 together with high levels of miR-221 and miR-744 implied poor prognosis in PC [58]. Although miRs have the advantages of small size, simple extraction, and convenient detection, they also have limitations that cannot be ignored including difficulty in quantification, dynamic changes, and susceptibility to interference. Therefore, a panel of multiple miRs is recommended for diagnosis to ensure the highest reliability and accuracy.

### Long non-coding RNAs (lncRNAs)

LncRNAs refer to non-coding RNAs with a length of over 200 nt. They are transcribed from intergenic and intronic regions in the genome by RNA polymerase II, and are involved in transcriptional and epigenetic regulation, pre- and post-translational regulation, cell cycling, differentiation, and other vital cellular activities. Tahira et al. [102] first explored lncRNA profiling in PC and found differential expression of lncRNAs in primary and metastatic PC lesions by using a cDNA microarray platform. Wang et al. [103] reported that the lncRNAs, HOTTIP-005 and RP11-567G11.1, and their plasma/serum fragments (HDRF and RDRF) were promising prognostic and diagnostic biomarkers of PC. Liu et al. [104] screened and validated three distinctive candidate lncRNAs composed of ABHD11-AS1, LINC00176 and SNHG11 for the diagnosis of PC. Among them, ABHD11-AS1 had the best diagnostic ability with higher diagnostic sensitivity than CA19-9, CEA and CA125, and combined with CA 19-9, ABHD11-AS1 was more effective than using it alone. The lncRNAs are very promising diagnostic tools, but more studies on their relationship to PC are required to optimize their use in the clinic.

### Exosomes (EVs)

Exosomes (EVs) are disc-shaped vesicles with diameters of 40–100 nm. They are produced by many types of cells including cancer cells, and subsequently released through the plasma membrane into body fluids, such as blood, saliva, urine, and cerebrospinal fluid. EVs appear to be involved in the initiation, development and metastasis of PC [105]. Multiple tissue-specific components such as pathogenic mRNAs, miRNAs, DNA fragments and proteins are present in the exosome’s lipid bilayer membrane, which suggests that it is possible to find candidate markers in EVs for diagnosis, prediction and prognosis of PC. The overexpression of several proteins has been reported in PC-derived exosomes. Among these proteins, glypican-1 (GPC1) in PC exosomes can differentiate PC patients from those with benign pancreatic disease and healthy controls with almost perfect accuracy (AUC = 1.0) and higher sensitivity and specificity than that in whole serum [32]. The panel of miR-10b, -21, -30c, -181a, and -let7a also showed good PC diagnostic accuracy [99]. The DNA contained in EVs also has high diagnostic value for PC because of the presence of high-frequency somatic mutations, copy number variations (CNVs) and gene fusions. Allenson et al. compared the incidence of KRAS mutation in exosome-derived DNA (exoDNA) and cfDNA from 263 individuals (127 PDAC patients and 136 controls). The results showed a higher detection rate of patients with localized PDAC by screening exoDNA for KRAS mutations. Furthermore, 43.6% of early-stage PC patients with mutated KRAS exoDNA were detected, indicating the promising capability of exoDNA for early PC diagnosis. In terms of prognosis, exosomal integrin αvβ5 could be a useful indicator for predicting distant metastasis because of the higher levels in PC patients with liver metastases compared to non-metastatic or healthy individuals. Exosomal α6β4 and α6β1 have also been shown to be associated with lung metastasis [105]. In addition, another study evaluated the prognostic role of macrophage migration inhibitory factor (MIF) in exosomes from PC patients and found that there was a significant increase in MIF levels in stage I PC patients who later developed liver metastasis [106]. Those studies investigated the role of exosomes as a prognostic biomarker in predicting distant metastasis.

In general, most studies have focused on evaluating the diagnostic and prognostic roles of exosomes in PC patients and healthy populations. However, the predictive role of exosome in PC treatment and its early diagnostic role in different stages of PC precancerous lesions are equally important and should be further explored in the future. Although the detection of EVs has unique advantages, such as widespread presence in various body fluids and relatively stable long-term storage at −80°C, the difficulty of isolation and purification currently limits their clinical application as potential biomarkers, but future research advances should improve the techniques.

## Metabolism-Related Biomarkers

Cellular metabolism is a common term for the series of ordered chemical reactions that occur in cells to maintain life activities. However, normal metabolism is not sufficient to meet the needs of tumorigenesis and tumor development. Tumor tissue can undergo an extensive metabolic reprogramming, which provides the biological basis and energy guarantee for cell proliferation, migration, and evasion of immune surveillance. Understanding abnormal tumor metabolism has also become one of the top ten goals of cancer researchers. Because there are extensive metabolic reprogramming pathways in PC, the detection of specific products of these pathways, such as carbohydrates, amino acids and lipids, is of great importance in the diagnosis and prognosis of PDAC.

Metabolomics is a technique for the quantitative analysis of all metabolites in an organism, which are mostly small molecules with molecular weights <1 kDa. The technology now exists to detect and characterize these small cancer-related abnormal products and intermediates from tumor metabolic reprogramming to achieve a specific diagnosis of cancer [107]. Sugimoto et al. [108] identified 57 metabolites associated with defined diseases in 251 individuals (69 oral, 18 pancreatic, 30 breast cancer patients, 11 persons with periodontal disease, and 87 healthy individuals) and 48 metabolites were identified as candidate biomarkers for PC. Another study examining serum samples from 43 PC patients and 42 controls showed that metabolite profiling was significantly more accurate than the conventional biomarkers, CA19-9 and CEA, (AUC = 0.92857, 0.82420 and 0.79956, respectively). Metabolomics also showed comparable diagnostic sensitivity (86.0%) and specificity (88.1%) to conventional markers. In addition, metabolomics products have also been proven to be useful in distinguishing PC patients with intermediate and advanced pathological stages. For example, some biomarkers identified for the diagnosis of early PDAC include palmitic acid, glucitol, xylitol, and inositol. Mayerle et al. [109] performed metabolomics testing on 914 subjects and identified a metabolic panel composed of nine components: histidine, proline, sphingomyelin d18:2, sphingomyelin d17:1, phosphatidylcholine, isocitrate, sphingosine-1-phosphate, pyruvate, and ceramide. This panel showed higher accuracy for the diagnosis of PDAC when combined with CA19-9. These results suggest that a combination of specific metabolites with CA19-9 can be used to provide higher diagnostic accuracy of PDAC.

The detection and profiling of amino acid metabolites has also been reported. Studies have shown that the concentrations of most amino acids are significantly different between PC patients, persons with pancreatitis, and healthy controls. For example, compared with controls, there was a significant increase in serine, but a significant decrease in the levels of threonine, asparagine, proline, alanine, citrulline, valine, methionine, leucine, tyrosine, phenylalanine, histidine, tryptophan, lysine and arginine in PC patients (*p* < 0.05). Another study, involving 40 PC patients, 23 pancreatitis patients, and 40 healthy controls determined amino acid profiles in serum samples by tandem mass spectrometry (TMS) and multi-marker models. The results showed that the diagnostic accuracy of the combined amino acids panel was much better than that of CA19-9 alone (AUC: 0.891 *vs.* 0.528). In conclusion, amino acid metabolites can be used as diagnostic markers for PC and pancreatic lesions, but more studies are still needed to validate the findings. The stability and accuracy of the assay could also be improved at a later stage by incorporating the measurement of amino acid ratios. In the area of lipid metabolites, a specific group of free fatty acids was shown to be reduced in PC, and there was also an alteration in the tumor’s lipid metabolic network which included PNLIP, CLPS, PNLIPRP1/2 and other key lipolytic enzymes. Therefore, the detection of lipid metabolites also has diagnostic value.

Metabolites may also be used as predictive/prognostic biomarkers in PC by employing dose-matching/escalation models, gas chromatography-mass spectrometry (GC/MS), and stable-based dynamic metabolic profiling (SiDMAP). Harris et al. conducted a metabolomic study on the role of luteolin, resveratrol, quercetin, and C75 (a fatty acid synthase inhibitor) on PaCa-2 cells. The results showed that luteolin could regulate fatty acid and nucleic acid synthesis together with energy production and further inhibit cell proliferation. Cantoria et al. [110] used PaCa-2 cells and targeted tracers coupled with GC/MS and found that the synthesis of fatty acids and cell membranes in PaCa-2 cells could be synergistically inhibited by metformin, mutant KRAS, and high cholesterol together.

To sum up, an integrated metabolic network formed by the complex connections and regulatory nodes exists among the multifarious metabolic processes. Considering the numerous enzymes and intermediates that can influence the concentration of a single metabolite, and lead to nonlinear quantitative relationships in concentration curves, it may be necessary to develop a panel of metabolic biomarkers to achieve an efficient and robust diagnosis of PC.

## Pancreatic Cancer Stem Cells (PCSCs)

Cancer stem cells (CSCs) are a group of cancer cells with the stem cell-like characteristics of self-renewal and multi-lineage differentiation potential. This self-renewal property is the main reason for tumor recurrence, metastasis and poor prognosis [111]; therefore, biomarkers for identifying CSCs in treated PC patients can be used to determine prognosis. Many stem cell markers, such as CD44, CD24, ESA, CD133, DCLK1, ALDH1, ALDHB1 and c-Met, are widely utilized in isolating and characterizing CSC populations. However, only a few known surface markers are expressed on PCSCs, and there are no unique markers that have been identified to isolate CSCs from different tumor types. Therefore, combinations of several markers are often used to improve the purity of isolated CSCs.

**Figure 1:**
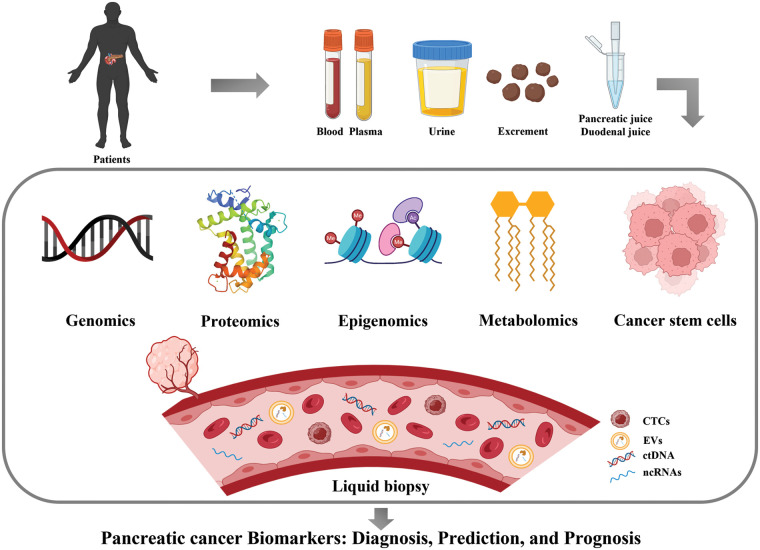
Major biomarkers of pancreatic cancer (Drawn by BioRender).

Expression of CD44, CD24 and ESA was first used to identify PCSC populations. Compared to cells with negative expression of each marker, PC cells characterized by CD44^+^CD24^+^ESA^+^ exhibited high self-renewal ability and resistance to chemoradiotherapy and showed a 100-fold increase in tumorigenesis. CD133 (prominin-1) is a glycosylated protein that was originally developed as a natural hematopoietic stem cell marker and was later discovered to identify CSCs. Hermann et al. analyzed PDAC cell lines and 11 primary human PDAC samples, and the CD133^+^ subsets were defined as CSCs with stem cell-like properties [112]. The CD133^+^ patients showed a lower 5-year survival rate than CD133^−^ patients (*p* = 0.0002) and CD133 as an independent prognostic factor was confirmed by multivariate analysis (*p* = 0.0103) [113]. Doublecortin-like kinase 1 (DCLK1) is a new PCSC marker that exists in normal pancreatic duct and islet cells, PanINs and PDAC. Its overexpression increased tumorigenicity and was associated with a shorter survival rate [114]. Aldehyde dehydrogenase-1 (ALDH1) was also regarded as a stem-cell marker. ALDH1^+^ cells isolated from pancreatic tumors have a mesenchymal phenotype, which is associated with high aggressiveness, low survival and high metastasis. However, ALDH1 was also highly expressed in normal pancreatic tissues, which means that ALDH1 alone cannot be used to identify CSCs [115]. The expression of hepatocyte growth factor receptor (c-Met) can also be used to identify PCSCs as well as high levels of CD44 [116]. The leucine-rich G-protein-coupled receptor 5 (LGR5), a Wnt-targeted gene, was also considered as participating in the activation of stem cell function. LGR5 was expressed in resected PDAC tissues and LGR5-positive patients exhibited shorter median survival rates [117]. These results suggested that LGR5 may also be useful as a potential marker of PCSCs. Taken together, the identification of PCSCs through markers is highly significant for determining patient prognosis and guiding the therapeutic regimen. Pancreatic cancer biomarkers in this article are shown in Fig. 1 and Table 2.

**Table 2:** Selected biomarkers for pancreatic cancer

Category	Biomarkers	Sample source	Application
Proteomic biomarkers	CA 19-9, CA 50, CA 242, CA 125	Serum	Diagnosis
	CEACAM5, CEACAM1,	Serum	Diagnosis, Prognosis
	MUCs	Tumor tissue, cystic fluid	Diagnosis, Prognosis
	RTKs (EGFR, IGFBP, VEGFA)	Serum	Diagnosis, Prognosis, Prediction
	MIC-1	Serum	Diagnosis, Prognosis
	GPC1	Serum	Diagnosis, Prognosis
	OPN	Serum	Diagnosis
	SPARC	Tumor tissue	Prognosis, Prediction
	S100A2, S100A6, S100P	Tumor tissue, duodenal juice	Diagnosis, Prognosis
	Tu M2-PK	Serum, plasma	Diagnosis, Prognosis
Genetic markers	*KRAS*	Fluid, tumor tissue	Prognosis
	*TP53*	Tumor tissue, duodenal fluid	Diagnosis
	*CDKN2A*	Tumor tissue	Prognosis
	*SMAD4*	Tumor tissue	Prognosis, Prediction
	*BRCA1/BRCA2*	Tumor tissue	Prognosis, Prediction
	*RNF43*	Tumor tissue, cyst fluid	Diagnosis
	*GNAS*	Serum	Diagnosis
	*GATA6*	Tumor tissue	Diagnosis, Prognosis, Prediction
	*HER2*	Tumor tissue	Prognosis
	*BRAF* ^*V600E*^	Tumor tissue	Prognosis, Prediction
Epigenetic markers	DNA methylation (*GNMT, MUC4, UCHL1, CLDN5, NPTX2* and *SFRP1*, *CLDN4, LCN2, 14-3-3r (SFN), TFF2, S100A4* and *MSLN*)	Tumor tissue	Diagnosis, Prognosis
	H3K27me3	Tumor tissue	Prognosis, Prediction
Metabolism-related biomarkers	Carbohydrates, lipids, amino acids	Serum	Diagnosis, Prognosis, Prediction
PCSCs markers	CD44, CD24, ESA, CD133, DCLK1, ALDH1, LGR5 and c-Met	Serum	Diagnosis, Prognosis
Liquid biopsy	CTCs (epithelial markers, mRNA and DNA mutations)	Peripheral blood	Diagnosis, Prognosis
	ctDNA (somatic mutations, CNVs, etc)	Plasma	Diagnosis, Prognosis, Prediction
	ncRNAs	Peripheral blood, urine, stool	Diagnosis, Prognosis
	Exosomes (mutant DNA, mRNA, miRNA and proteins)	Blood and other fluid	Diagnosis, Prognosis

## Conclusions and Future Perspectives

Pancreatic cancer is a malignant disease with a high recurrence rate and mortality. Insidious onset, lack of early symptoms, vague atypical symptoms, deep anatomical position, complex pathogenesis, and lack of non-invasive tests for extensive screening make an early diagnosis of PC difficult, which means that in most PC patients can only carry out exploratory or palliative surgery when the disease is diagnosed. Therefore, it is of crucial importance to discover and evaluate key biomarkers of pancreatic cancer.

With the advancement of technology, the detection of biomarkers from serum, plasma, pancreatic fluid, urine and feces is considered to be an effective, stable and safe alternative to traditional surgical specimens and tissue biopsies. Biomarkers from body fluids, such as pancreatic-specific proteins, genetic and epigenetic markers, metabolites and tumor stem cells, have great diagnostic value for accurately distinguishing pancreatic cancer from other diseases. Relevant markers can also be used to predict the prognosis of patients and guide the therapeutic treatment with appropriate drugs. Liquid biopsies are gradually taking over the focus of accurate diagnosis and treatment of tumors, because they are non-invasive and can be repeated at different stages of disease treatment. Nevertheless, the usefulness of some biomarkers in PC is still controversial, so more research is needed to determine the optimal combination of different marker panels.

It is noteworthy that there can be false positive or negative results in the detection of a single biomarker, which confirms the necessity of developing an optimal diagnostic panel of biomarkers. Therefore, the combination of multiple biomarkers or multiple imaging technologies (such as CT/MRI/EUS) is undoubtedly the future trend for early diagnosis and a better prognosis of pancreatic cancer.
